# Resveratrol Increases Glucose Induced GLP-1 Secretion in Mice: A Mechanism which Contributes to the Glycemic Control

**DOI:** 10.1371/journal.pone.0020700

**Published:** 2011-06-06

**Authors:** Thi-Mai Anh Dao, Aurélie Waget, Pascale Klopp, Matteo Serino, Christelle Vachoux, Laurent Pechere, Daniel J. Drucker, Serge Champion, Sylvain Barthélemy, Yves Barra, Rémy Burcelin, Eric Sérée

**Affiliations:** 1 Institut National de la Santé et de la Recherche Médicale U1048, Institut de recherche sur les Maladies Métaboliques et Cardiovasculaire, I2MC, Toulouse, France; 2 Université de Toulouse, UPS, Institut des Maladies Métaboliques et Cardiovasculaires (I2 MC), Hôpital de Rangueil, Toulouse, France; 3 Institut National de la Recherche Agronomique 1260, Faculté de Pharmacie, Marseille, France; 4 ENTERONOVA SAS, Incubateur Midi-Pyrennées, Toulouse, France; 5 Department of Medicine, Samuel Lunenfeld Research Institute, Mount Sinai Hospital, University of Toronto, Toronto, Ontario, Canada; 6 YVERY SARL, Marseille, France; University of Bremen, Germany

## Abstract

Resveratrol (RSV) is a potent anti-diabetic agent when used at high doses. However, the direct targets primarily responsible for the beneficial actions of RSV remain unclear. We used a formulation that increases oral bioavailability to assess the mechanisms involved in the glucoregulatory action of RSV in high-fat diet (HFD)-fed diabetic wild type mice. Administration of RSV for 5 weeks reduced the development of glucose intolerance, and increased portal vein concentrations of both Glucagon-like peptid-1 (GLP-1) and insulin, and intestinal content of active GLP-1. This was associated with increased levels of colonic proglucagon mRNA transcripts. RSV-mediated glucoregulation required a functional GLP-1 receptor (Glp1r) as neither glucose nor insulin levels were modulated in Glp1r-/- mice. Conversely, levels of active GLP-1 and control of glycemia were further improved when the Dipeptidyl peptidase-4 (DPP-4) inhibitor sitagliptin was co-administered with RSV. In addition, RSV treatment modified gut microbiota and decreased the inflammatory status of mice. Our data suggest that RSV exerts its actions in part through modulation of the enteroendocrine axis *in vivo*.

## Introduction

Type 2 diabetes (T2D), classically arises as a result of defects in insulin secretion and insulin action. Considerable evidence suggests that low-grade inflammation may also exacerbate metabolic control by impairing insulin action and secretion [1]. In the quest of a unifying molecular mechanism, impaired mitochondrial metabolism has been linked to inflammation [2]. Increased inflammation is also associated with impaired adipose tissue physiology [3] which has been recently linked to a change in intestinal microbiota and lipopolysaccharide production [4], [5]. Our current concepts of how existing anti-diabetic agents exert their mechanisms of action continue to evolve, as exemplified by studies of the biguanide metformin. Recently new mechanisms of action of this well-known biguanide have been described that encompass enhanced secretion and action of Glucagon-like peptid-1 (GLP-1) [6], [7] a gut hormone which increases insulin secretion [8], [9], [10] . This seems to make metformin an ideal oral antidiabetic agent for use alone, or in combination with other agents that exert their glucoregulatory effects through complementary mechanisms of action.

Resveratrol (RSV) is a natural phytoalexin (3,4′,5-trihydroxytrans-stilbene) produced by various plants such as the red grapes (*Vitis vinifera* L.), peanuts (*Arachis* spp), berries (*Vaccinium* sp), and *polygonum cuspidatum*, that exerts multiple beneficial metabolic actions in vivo [11], [12], [13]. Resveratrol is known to be a strong antioxidant and possesses anti inflammatory properties [14], [15]. It inhibits NFκB- and AP-1-dependent inflammatory processes, resulting in reduction of levels of IL-1, TNFα and other inflammatory cytokines. Over the last decade several mechanisms have been proposed to explain the glucoregulatory actions of RSV. This polyphenol has been shown to enhance Sirtuin-1 (SIRT1) activity and to improve insulin secretion [16], [17] and sensitivity [18], [19], increase mitochondrial number and function [12], [20], [21], decrease adiposity, reduce glucose, and prolong life of mice fed a calorie enriched diet [13], [22]. However, since the central role of SIRT in these beneficial actions is, to date, controversial [23], the direct targets of RSV remain unclear.

Recently, our laboratory has shown that diabetic mice treated with Benzopyren, an aryl hydrocarbon receptor (AhR) agonist, exhibit reduced GLP1 secretion [24]. As RSV is also an antagonist of AhR [25], we hypothesized that RSV might trigger GLP-1 secretion and improve glycemia. Our results show that a five week chronic treatment with RSV is associated with increased circulating levels of GLP-1 and insulin and enhanced levels of intestinal proglucagon mRNA transcripts. Consistent with these findings, RSV combined with a Dipeptidyl peptidase (DPP-4) inhibitor augments portal GLP-1 concentrations and further improves glucose homeostasis. The glucoregulatory actions of RSV are abolished in GLP-1 receptor knockout (Glp1r-/-) mice and associated with increased levels of anti-inflammatory IL-10 cytokine expression and changes in gut flora of diabetic mice. These findings expand our concepts of how RSV exerts its metabolic effects to encompass activation of the enteroendocrine system and control of glycemia through GLP-1-receptor-dependent mechanisms of action.

## Materials and Methods

### RSV formulation and dosage

The natural purified trans-Resveratrol is formulated with polysorbate 20, and polyglyceryl-3Dioleate (Yvery, France). The RSV was daily mixed with the diet for animal experiments at the dose of 60 mg RSV/Kg/day.

### Animal and treatment

Eight week-old male C57Bl/6J wild type mice (Charles River, L'Arbresle, France) and Glp1r^−/−^ mice from our colony (in C57Bl/6 background) were housed in a specific pathogen-free condition with a 12-/12-hour light (10 p.m.)/dark (10 a.m.) cycle and had free access to water and food. Mice were maintained on normal chow diet (NC, energy content: 12% fat, 28% protein, and 60% carbohydrate), or a high-fat diet (HFD; energy content: roughly 72% fat comprising corn oil and lard, 28% protein, and <1% carbohydrate, SAFE, Augy, France) for five weeks. This diet induces diabetes before the onset of obesity [4], [5], [26], [27]. A subset of mice was treated with the fat-enriched diet supplemented with RSV. In addition, another group of mice was treated with RSV and a DPP-4 inhibitor, sitagliptin (Januvia®, Merck Sharp and Dohme-Chibret, France) (5 mg/day, in the food). Food intake, body weight, and glucose tolerance were measured as previously described [28]. All animal experimental procedures were approved by the local animal ethical committee of the Rangueil hospital under the authorization number “31–278”.

### Oral glucose tolerance test and insulin assays

An oral glucose tolerance test (OGTT, 2 g/kg of glucose) was performed in 6 h-fasted mice after five weeks of treatment. Blood glucose concentrations were monitored from the tip of the tail vein with a glucose meter (Roche Diagnostic, Meylan, France) at −30, 0, 30, 60, 90 and 120 min after oral glucose administration, as previously described [28]. Area under the curve (AUC) (30–90) was calculated for each group of mice. Plasma insulin concentration was determined by ELISA (Mercodia, Uppsala, Sweden) by using 10 µl of plasma from normal chow and HFD +/− RSV treated mice.

### GLP-1 measurement in portal plasma and colon

For plasma portal GLP-1 quantification, mice (in fed state) were rapidly anesthetized by intra-peritoneal injection (0.1 ml/10 mg body weight) of Ketamine (Vibrac, France) and Xylazine hydrochloride 2% Rompun® (Bayer, France) in sodium chloride (0.9%; 2∶1∶7 v/v/v), dissected and the portal blood samples were collected in EDTA tubes (Sarstedt, Numbrecht, Germany) containing a DPP-4 inhibitor (Linco Research, St Charles, MO, USA). Following sacrifice, segments of colon were immediately excised, immersed in liquid N_2_ and stored at −80°C for further mRNA and peptide analyses.

For assessment of levels of colonic GLP-1, intestinal samples were homogenized in ethanol/acid (100% ethanol: sterile water: 12N HCl 74∶25∶1 v/v) solution (5 ml/g tissue). Then the homogenates were centrifuged (2000 g for 20 minutes) and supernatants were collected and diluted 50-fold. Concentrations of GLP-1 (7–36) amide were determined using an ELISA method (Glucagon-Like-Peptide-1 active ELISA kit, Millipore, France).

### RNA extraction and real time PCR

Total RNA was isolated from tissues using Trizol reagent (Invitrogen, France) and quantified by NanoDrop (NanoDrop technologies Inc., France). Total RNA (1 µg) was reverse-transcribed using Moloney murine leukemia virus reverse-transcriptase (Invitrogen, Cergy-Pontoise, France) and random primers at 42°C for 1 h. The expression of target genes was determined using the Stratagene Mx 3005p. The mRNA concentration of target genes was normalized to levels of β2-actin mRNA and the results were expressed as relative expression levels (REL). The data were quantified by the method of 2^-ΔΔCt^. Primers used are listed in table 1.

**Table 1:** Primers Used.

Genes	Forward sequence (5′-3′)	Reverse sequence (5′-3′)
β2-actin	5′-AAGGCCAACCGTGAAAAGAT-3′	5′-GTGGTACGACCAGAGGCATAC-3′
TGF-β	5′-TGGAGCAACATGTGGAACTG-3′	5′-GTCAGCAGCCGGTTACCA-3′
IL-10	5′-CACAAAGCAGCCTTGCAGAA-3′	5′-AGAGCAGGCAGCATAGCAGTG-3′
TNFalpha	5′-TGGGACAGTGACCTGGACTGT-3′	5′-TTCGGAAAGCCCATTTGAGT-3′
Proglucagon	5′-GACATGCTGAAGGGACCTTTAC-3′	5′-GGCTTTCACCAGCCAC-3′
V3 16S rDNA universal	5′-GCCCGGGGCGCGCCCCGGGCGGGGCGGGGG CACGGGGGGACTCCTACGGGAGGCAGCAGT-3′	5′-GTATTACCGCGGCTGCTGGCAC-3′

### Determination of IL-10 protein concentration

Tissue protein extracts were obtained by homogenization of colonic segments (0.5 mg tissue/ml) in 50 mM Tris HCl, pH 7.4, 0.5 mM DTT and a cocktail of proteases inhibitors containing PMSF, ALI and POP (Sigma, France). Samples were centrifuged at 12,000 g for 10 minutes and stored at −80°C. IL-10 levels in colonic protein extracts were determined using an ELISA method (Mouse IL-10 ELISA Ready-SET-Go!, eBioscience, France).

### Intestinal microflora characterization

Total DNA was isolated from caecum using Trizol reagent (Invitrogen, France) and was amplified by PCR, targeting the V3 region of the 16S rRNA gene using the universal bacterial primers HDA1-GC and HDA2 (Table 1). Each reaction mixture (25 µl) contained 4 µl of DNA diluted to 50 ng/µl, deoxynucleoside triphosphate (Sigma-Aldrich – France) at a concentration of 200 mM, 0.3 µM of each primer, and 0.07 µl of *Taq* polymerase (Sigma-Aldrich – France). The following amplification program was used: 94°C for 5 min, 30 cycles consisting of 94°C for 30 s, 55°C for 45 s, and 72°C for 60 s, and 30 min at 72°C. Denaturing gradient gel electrophoresis (DGGE) was then performed by using DGGE 2401 systems (CBS & Scientific Co. – United State) and 8% polyacrylamide gels with a 35–55% gradient of urea (99.0–100.5% - Sigma-Aldrich-France) and formamide (99+% - Sigma-Aldrich-France), which increased in the direction of electrophoresis. Electrophoretic runs were in a Tris-acetate-EDTA buffer (40 mmol/l Tris, 20 mmol/l acetic acid, and 1 mmol/l EDTA) at 60 V and 60°C for 18 h. Gels were stained with SYBR Safe 1× (Invitrogen, France) for 30 min, rinsed with deionized water, then scanned and analyzed by using Typhoon 9400 Variable Mode Imager (Amersham Biosciences-United State). Hierarchical clustering was performed by using Permutmatrix 1.9.3.0 [29].

### Statistical Analysis

Results are expressed as means ± SEM. Statistical differences between groups were evaluated by one-way ANOVA followed by Tukey test and the non-paired –Student's T test using Sigma Stat 2.03. The level of significance was set at p<0.05.

## Results

### Effect of a five week treatment with RSV on HFD-induced glucose intolerance

**Figure 1:**
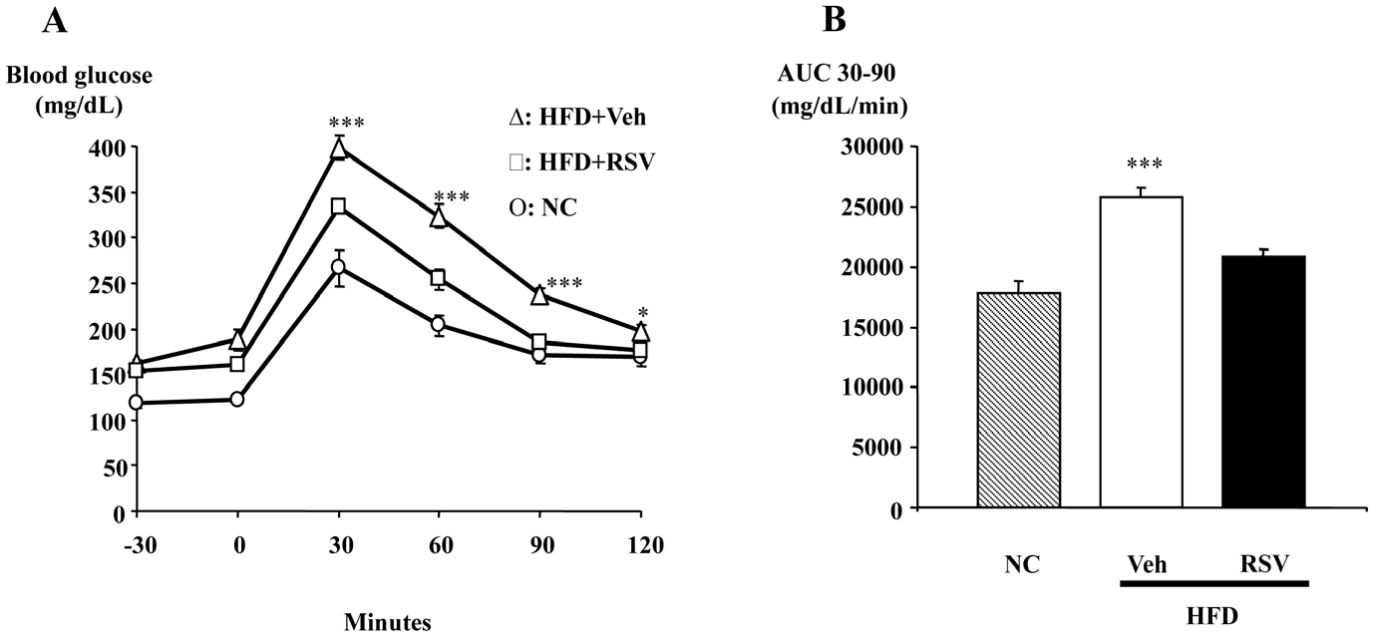
RSV improves glucose tolerance in high fat-fed diabetic mice. **A)** Glycemic profiles (mg/dL) of normal chow (circles), high fat diet-fed mice treated with vehicle (triangles) or RSV (squares) for five weeks and **B)** area under the curve for glucose (AUC); Data are presented as mean ± S.E.M, n = 8 mice per group * and *** statistically different between groups when p<0.05 and p<0.001, respectively, as analyzed by one-way ANOVA followed by Tukey test.

To assess the anti-diabetic effect of RSV, we treated HFD-diabetic mice with a dose of RSV, 60 mg/kg/day, for five weeks. RSV significantly reduced glucose intolerance in diabetic mice without affecting fasting glycemia (Figure 1A, B).

**Figure 2:**
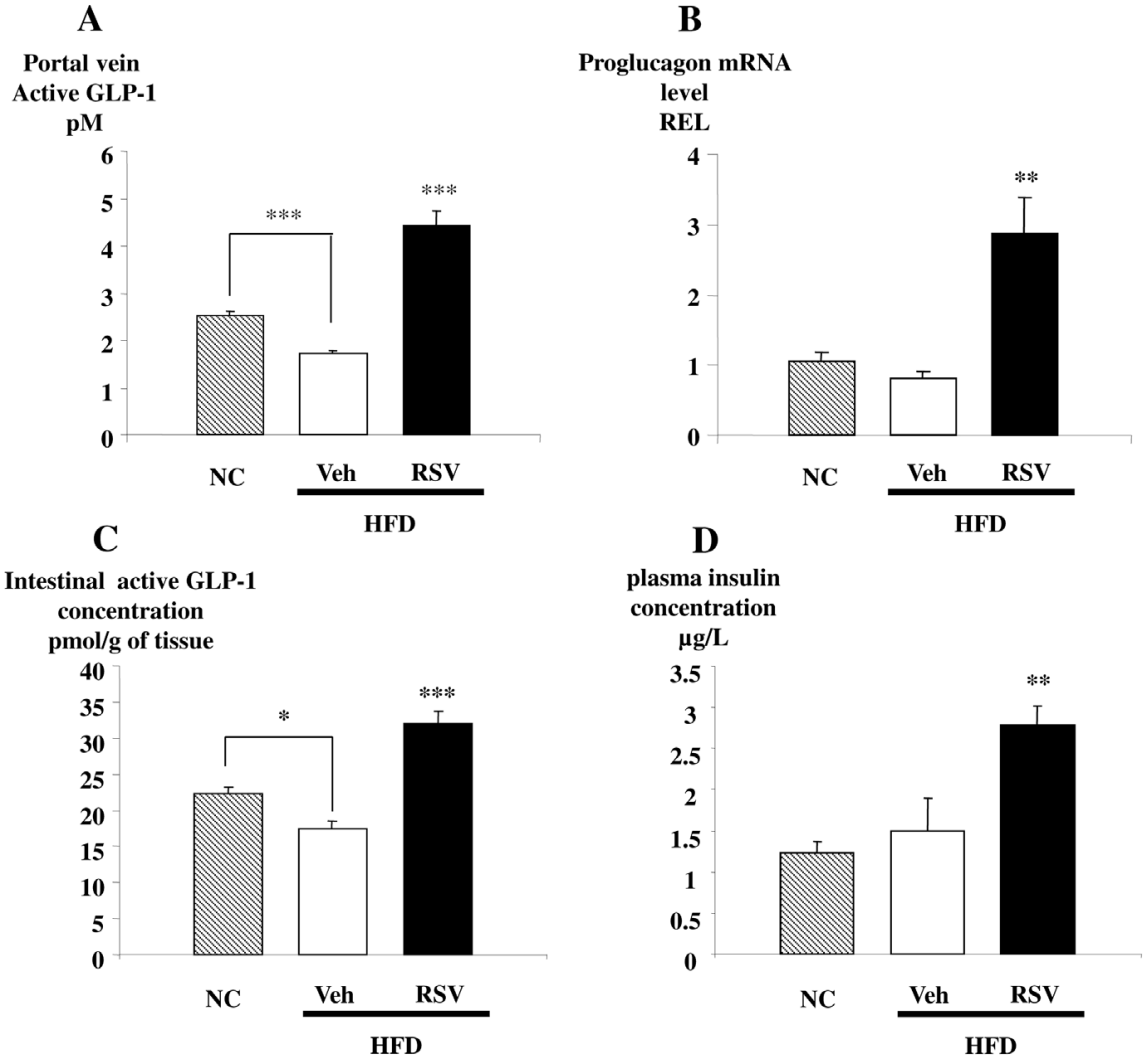
RSV increases levels of GLP-1 and Insulin. **A)** Portal vein active GLP-1 concentrations (pM); **B)** proglucagon mRNA concentration (Relative Expression Level, REL); **C)** intestinal GLP-1 concentrations (pmol/g of tissue) and **D)** portal plasma insulin concentrations (µg/L) of normal chow (stripe bars), high fat diet-fed mice treated with vehicle (open bars) or RSV (closed bars) for five weeks. Data are presented as mean ± S.E.M, n = 8 mice per group (in fed state) *, ** and *** statistically different between groups when p<0.05, p<0.01 and p<0.001, respectively, as analyzed by one-way ANOVA followed by Tukey test.

To understand the mechanisms mediating the pronounced salutary effects of RSV on oral glucose tolerance, we examined levels of GLP-1. Mice fed the high fat diet exhibited reduced levels of GLP-1 (Figure 2A), in contrast, RSV almost tripled the concentration of active GLP-1 in the portal vein (Figure 2A) and significantly increased the corresponding intestinal content of both proglucagon mRNA and active GLP-1 (3.4 and 1.8-fold, respectively, Figures 2B, C). Consistent with the change in GLP-1 levels, the plasma concentration of insulin was also significantly increased (1.8-fold) in response to the oral glucose challenge (Figure 2D).

### The glucoregulatory actions of RSV depend on a functional GLP-1 receptor and are further improved by a DPP-4 inhibitor

**Figure 3:**
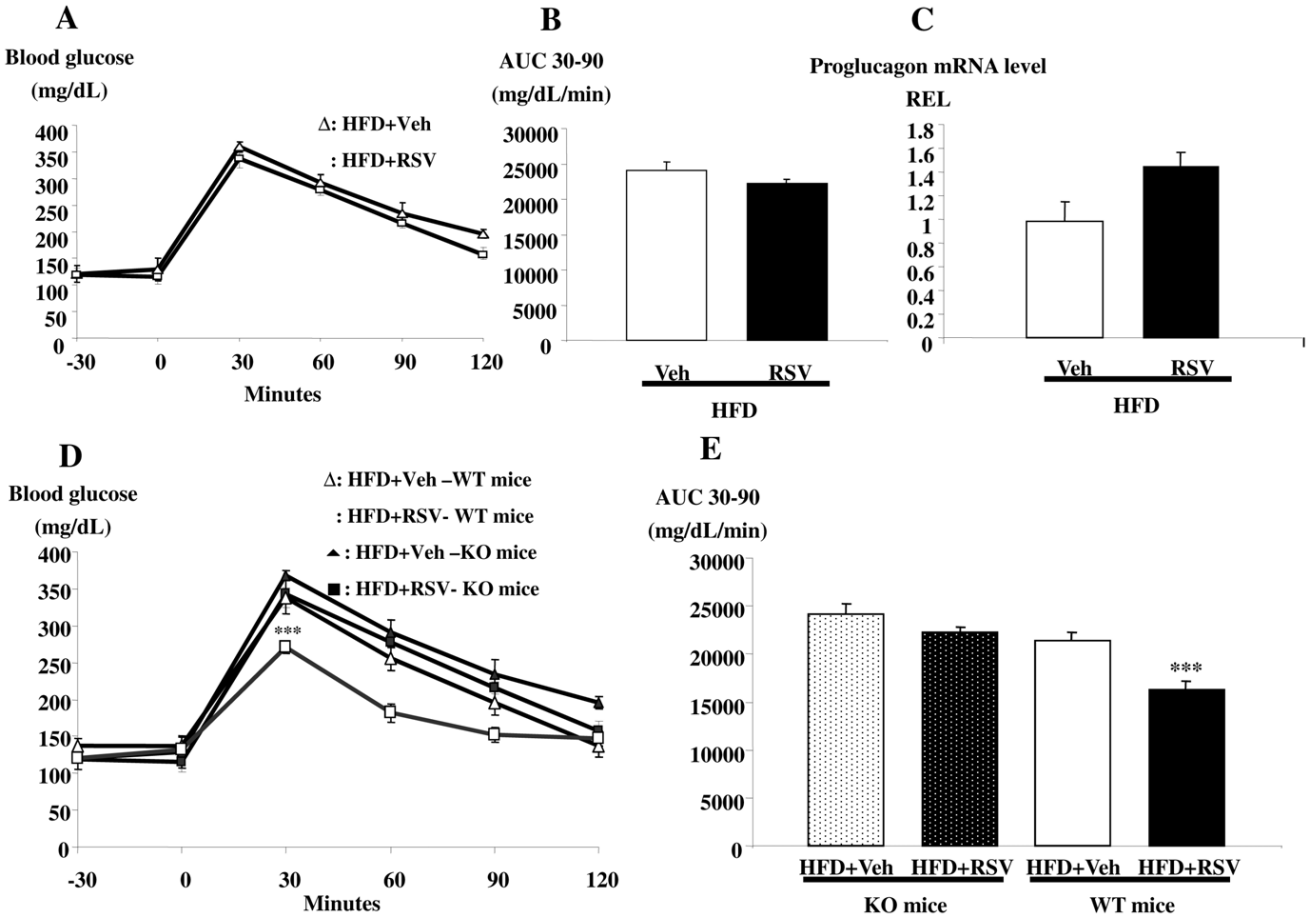
The glucose control by RSV is blunted in high fat diet-fed Glp1r^−/−^ mice. **A)** Glycemic profiles (mg/dL) of high fat diet-fed Glp1r^−/−^ mice treated with vehicle (triangles) or RSV (squares) for five weeks and **B)** an index of area under the curve glucose (AUC); **C)** proglucagon mRNA levels (Relative expression level REL) of high fat diet-fed mice treated with vehicle (open bars) and RSV (closed bars) for five weeks. **D)** Glycemic profiles (mg/dL) of high fat diet-fed wild type mice (high fat diet-fed mice treated with vehicule (white triangles) or RSV (white squares)) and Glp1r^−/−^ mice (high fat diet-fed mice treated with vehicule (black triangles) or RSV (black squares)) after five weeks of treatment and **E)** an index of area under the curve glucose (AUC). Data are presented as mean ± S.E.M, n = 8 mice per group.

To determine whether GLP-1 secretion and action mediated the improved glucose tolerance in response to the chronic RSV treatment, we analyzed oral glucose tolerance and GLP-1 concentrations in Glp1r^−/−^ mice. In contrast to data obtained with WT mice, Glp1r^−/−^ mice were insensitive to the RSV treatment revealing an essential role for the GLP-1R in control of glucose tolerance by RSV (Figures 3A, B). Furthermore, proglucagon mRNA levels were only modestly increased (1.3-fold) following RSV in Glp1r^−/−^ mice suggesting that the GLP-1 receptor was important for the regulated expression of its ligand (Figure 3C). We have compared the glycemic profile between wild type and Glp1r^−/−^ mice. The results demonstrated that improve of glucose tolerance was significantly different when wild type mice were treated with RSV (Figure 3D and 3E).

**Figure 4:**
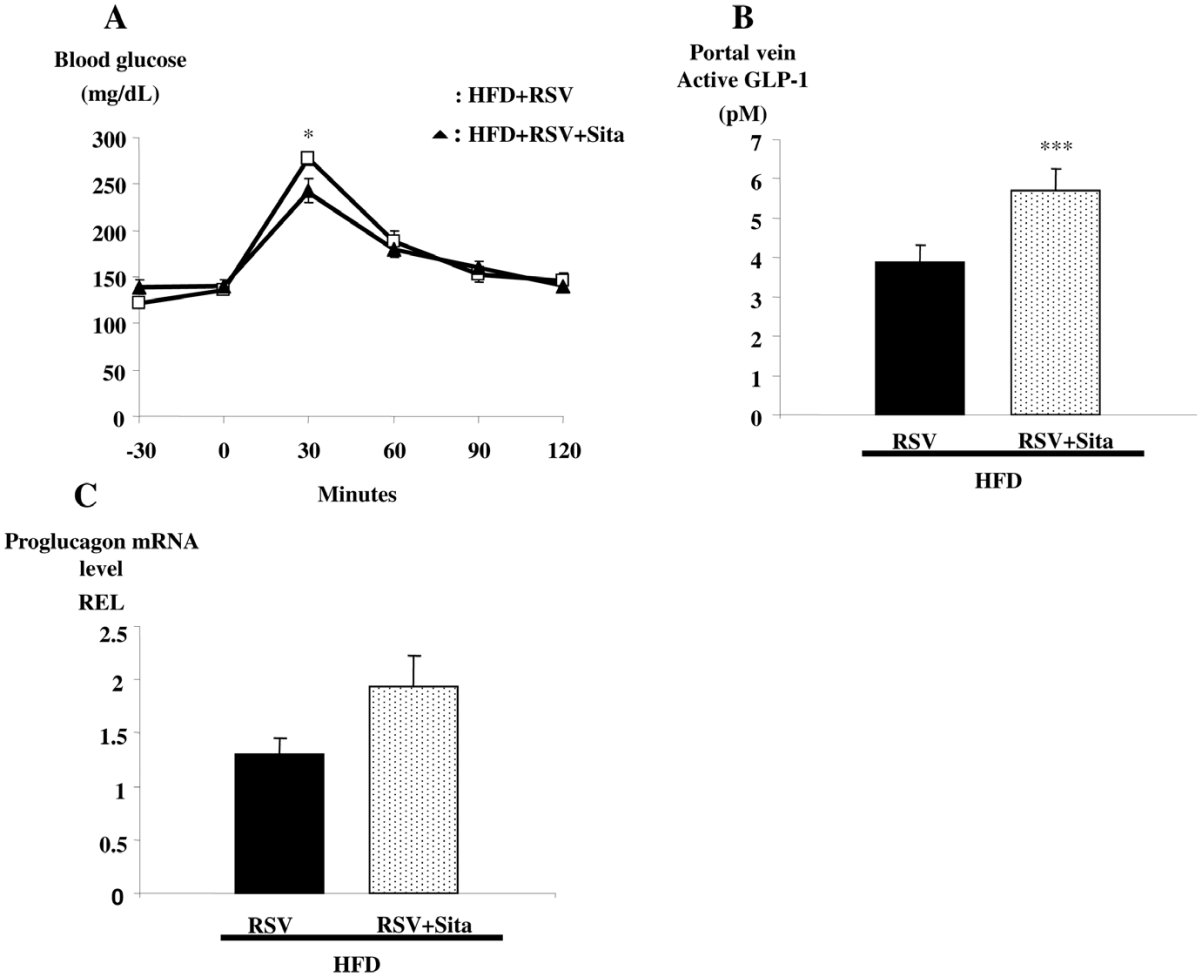
Co-administration of the dipeptidyl peptidase-4 inhibitor sitaglipin and RSV further improves glucose tolerance in high fat diet-fed diabetic mice. **A)** Glycemic profiles (mg/dL) of high fat diet-fed diabetic mice treated with RSV (squares), or RSV plus sitagliptin (triangles) for five weeks; **B)** portal vein active GLP-1 concentrations (pM) and **C)** proglucagon mRNA levels (Relative Expression Level REL) of high at diet-fed mice treated with RSV (closed bars) and sitagliptin plus RSV (spotted bars) for five weeks. Data are presented as mean ±S.E.M, n = 8 mice per group, * and *** statistically different between groups when p<0.05 and p<0.001, respectively, as analyzed by the Student's T test.

Next, we assessed whether the therapeutic efficacy of RSV could be further enhanced by potentiating levels of active GLP-1 through combination with a DPP-4 inhibitor. Oral glucose tolerance was further enhanced when the DPP-4 inhibitor, sitagliptin, was added to the RSV treatment (Figure 4A). Furthermore, the active GLP-1 concentrations were further increased (1.5-fold) in the portal blood (Figure 4B). However, the combined sitagliptin/RSV treatment did not significantly increase intestinal proglucagon gene expression when compared to administration of RSV alone (Figure 4C).

### Effect of a five week treatment with RSV on gut microbiota

**Figure 5:**
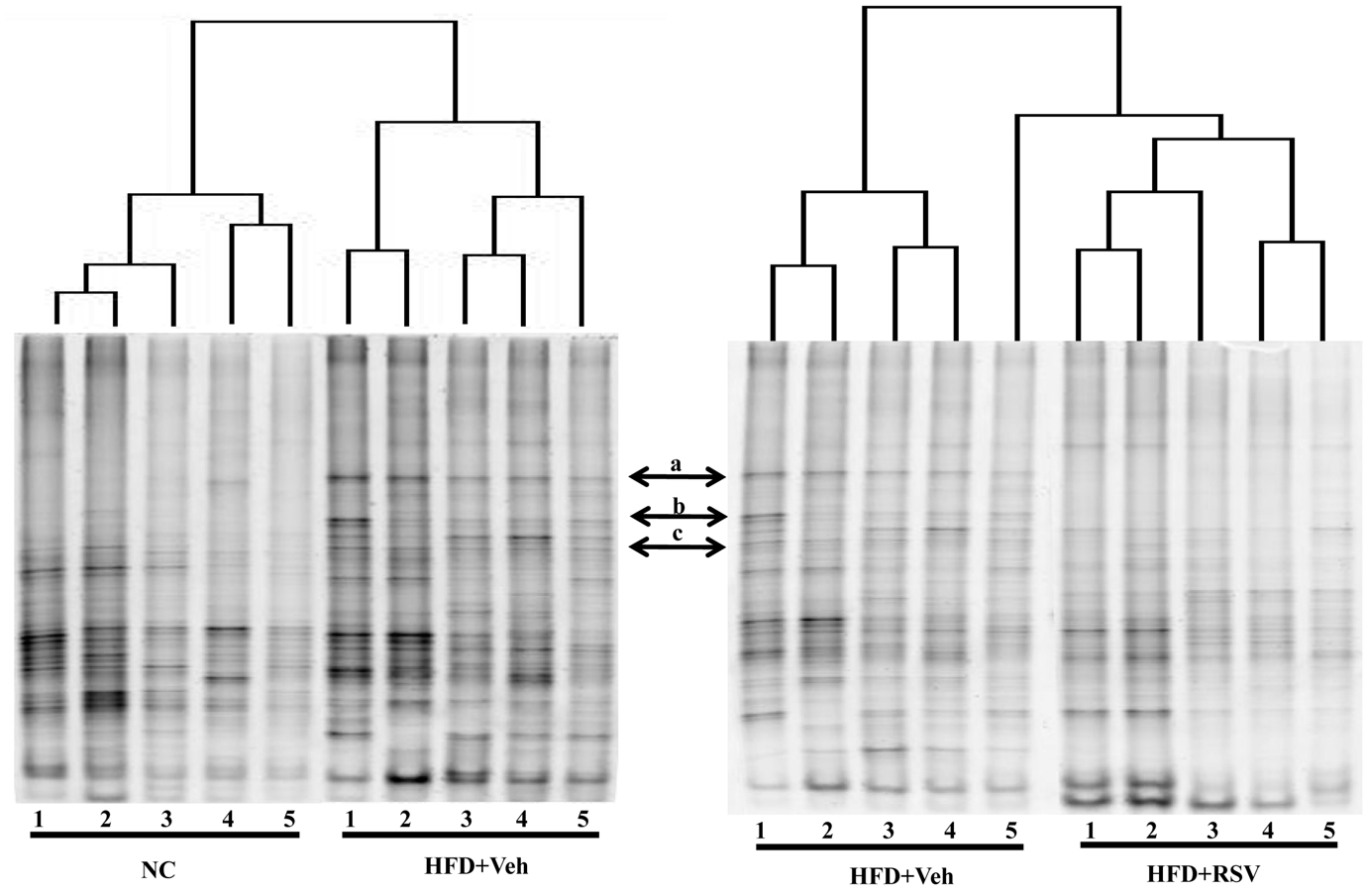
RSV has a prebiotic effect on gut microbiota. DGGE profiles generated from the caecal content of mice fed normal chow (NC), high fat diet and treated with vehicle (HFD±Veh), or RSV (HFD±RSV) for 5 weeks. Each number and profile corresponds to a different animal. The arrows denote a subset of bands, which have disappeared with the RSV treatment, were cloned and sequenced (see results for identification).

The above set of data suggested that RSV was targeting the intestine. Since RSV is known to be an antimicrobial agent [30], [31], [32], we determined whether the gut microbiota was also impacted by RSV treatment by using DGGE analyses. DGGE profiles clearly showed that after a five week treatment, RSV normalized the strongly modified caecal bacterial composition of animals fed a high-fat diet (Figure 5). Three of the bands found to be differently expressed between HFD-fed mice treated with or without RSV were sequenced and identified. They correspond to *Parabacteroides jonsonii DMS 18315* (a), *Alistipes putredinis DMS 17216* (b) and *Bacteroides vulgatus ATCC 8482* (c) All three bacteria were directly affected by RSV treatment (Figure 5, arrows a, b, c, respectively). In particular, these bands disappeared when mice were provided with RSV.

### Effect of a five week treatment with RSV on HFD-induced inflammation

**Figure 6:**
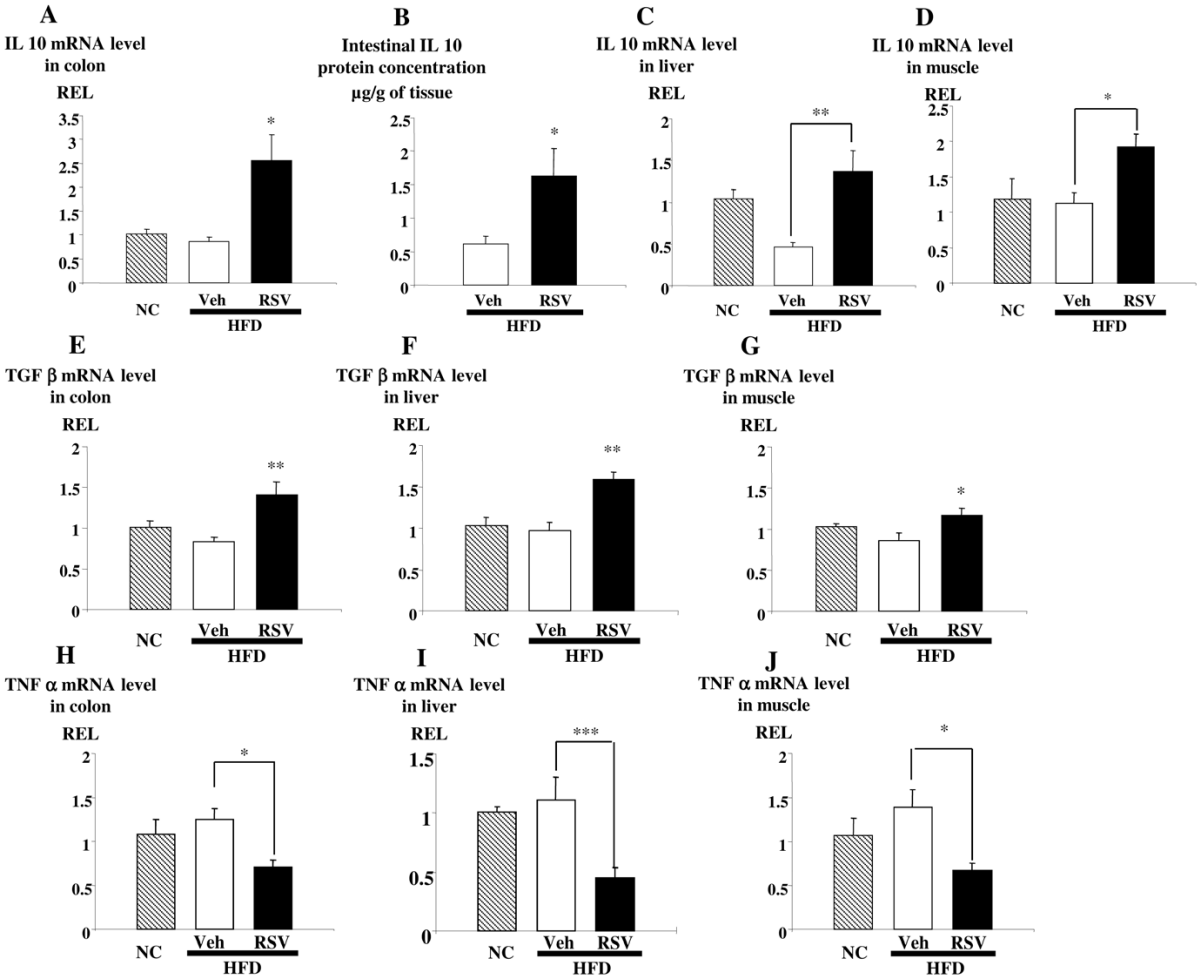
RSV decreases the inflammatory status in high fat-fed diabetic mice. IL-10 mRNA levels (Relative Expression Level, REL) **(A)** and IL-10 protein concentration (µg/g) **(B)** in colon; IL-10 mRNA in liver **(C)** and muscle **(D)**, TGF-β mRNA in colon **(E)**, liver **(F)**, muscle **(G)**, and TNF-α mRNA in colon **(H)**, liver **(I)**, muscle **(J)** of normal chow (stripe bars), high fat diet-fed mice treated with vehicle (open bars) or RSV (closed bars) for five weeks. Data are presented as mean ± S.E.M, n = 8 mice per group (in fed state) *, ** and *** statistically different between groups when p<0.05, p<0.01 and p<0.001, respectively, as analyzed by the Student's T test (Fig. 6B) and one-way ANOVA followed by Tukey test. (Fig. 6A, C, D, E, F, G, H, I, J).

Since changes in gut microbiota have been associated with the inflammatory status of metabolic diseases [4], [5] we evaluated the putative anti-inflammatory effect of RSV during a HFD treatment. RSV markedly increased IL-10 expression in the colon, liver, and muscle by 3.1, 3.7 and 1.7-fold, respectively (Figures 6A, B, C, D). TGF-β levels were also significantly increased in response to RSV (Figures 6E, F, G). Conversely, RSV induced a significant decrease of TNF-α expression in the same three tissues (Figures 6H, I, J). We have evaluated in brain the expression level of proglucagon, IL-10 and PAI-1. The results (data not shown) indicated that RSV did not modify the proglucagon mRNA level. In contrast, the PAI-1 mRNA level was decreased (3.5 fold) when RSV was added in HFD compared to HFD. IL-10 mRNA level significantly increase (2.1 fold) in HFD + RSV compared to HFD.

## Discussion

We here demonstrate that a chronic resveratrol treatment increases glucose-induced GLP-1 and insulin secretion. This mechanism was enhanced by a concomitant treatment with a DPP4 inhibitor and as a consequence altogether lowers glycemia of high-fat diet-induced diabetic mice. The putative GLP-1 dependency of resveratrol action was suggested since Glp1-/- mice were not sensitive to the treatment. The role of a change in intestinal microbiota and inflammation is also suspected.

Augmentation of GLP-1 action is now widely used for the treatment of T2D. Indeed, GLP-1 not only acts as an incretin to lower blood glucose via stimulation of insulin secretion from islet β cells but also exerts actions independent of insulin secretion, including inhibition of gastric emptying and acid secretion, reduction in food ingestion and glucagon secretion, and stimulation of β cell proliferation [8]. GLP-1 actions are highly glucose-dependent, hence GLP-1 administration is unlikely to be associated with hypoglycemia [33], a frequent side effect of many oral anti-diabetic agents and insulin. The only obstacle which prevents the native molecule to be used as a therapeutic agent for the treatment of diabetes is that GLP-1 is rapidly degraded within minutes by DPP-4 [34], [35], [36]. Consequently, stable GLP-1 receptor agonists (Liraglutide, Exenatide), and DPP-4 inhibitors (Sitagliptin, Vildagliptin, Saxagliptin, Alogliptin) have been developed for the treatment of diabetes.

Our current findings further extend the increasing number of agents known to exert their actions in part through enhancement of incretin activity by demonstrating that RSV given orally exerts an anti-diabetic effect linked to GLP-1 production. Indeed, oral glucose tolerance is improved by RSV in association with increased gut proglucagon gene expression and enhanced intestinal levels of GLP-1. Furthermore, these glucoregulatory actions of RSV are blunted in Glp1r^−/−^ mice. Although it is unlikely that all the anti-diabetic effect of resveratrol are mediated through the GLP-1 receptor our data strongly suggest that this new mechanism does represent a major mode of action in the high-fat diet-fed diabetic mouse. This hypothesis is further reinforced since the proglucagon gene expression in the gut was only moderately increased in Glp1r^−/−^ compared to RSV-treated wild type mice. This is in agreement with data showing that the portal levels of GLP-1 were reduced in RSV-treated Glp1r^−/−^ mice, suggesting that GLP-1 regulates the control of its secretion and gene expression [37]. Furthermore, our data demonstrate that co-administration of a DPP-4 inhibitor and RSV further enhanced the concentration of active portal GLP-1 and improved the glycemic control relative to that observed with the RSV formulation alone. This set of data provides a rationale for further studies examining the combinatorial efficacy of RSV and DPP-4 inhibition. This concept is consistent with strategies designed to enhance the efficacy of DPP4 inhibitors [38] and intriguingly metformin has also been shown to increase GLP-1 secretion through mechanisms, which are poorly understood [6]. On other hand, recent data showed that RSV at a very high dose also increases the plasma concentration of glucose-dependent insulinotropic peptide (GIP) [39]. This was associated with reduced body weight gain in a non-human primate model of obesity [39]. Although we observed increased GIP mRNA levels in the intestine (data not shown), the significantly diminished glucoregulatory activity of RSV in Glp1r^−/−^ mice suggests that most of the therapeutic effects of RSV in our experimental model are mediated by GLP-1.

It has been previously described that RSV crosses the blood brain barrier and can have an effect on the central nervous system (CNS) [40], [41]. The pharmacological actions of RSV on the CNS can be the consequence of an antioxidant and anti-inflammatory activity, and on the proglucagon level. We have evaluated the proglucagon level in the brain of the animals. Our results indicated (data not shown) that RSV does not induce the expression of GLP-1 in hypothalamus. However, we did observed a slight increase in IL10 mRNA concentration and a reduction of PAI 1 mRNA concentration in the hypothalamus suggesting that some anti-inflammatory effect of resveratol could be suspected. With these later set of data we cannot rule out that part of the anti-diabetic effect of reseveratrol might be through a central beneficial regulation.

In peripheral organs our present data show that RSV reduces inflammation in part through enhancement of IL-10 production in colon, liver and muscle. In addition, this effect was associated with a decrease of TNF-α mRNA levels and a favorable modulation of intestinal microbiota, which might be linked to IL-10 synthesis in these three tissues. Inflammation induced by the infusion of bacterial lipopolysaccharides reduced glucose-induced insulin secretion and led to insulin resistance [4], and increase production of cytokines through a mechanism requiring the LPS receptor CD14. Similarly, the inflammatory status induced by the change of microbiota might contribute to the impairment of GLP-1 secretion in mice on a HFD diet. We previously showed that prebiotic treatment reverted the alteration of intestinal microbiota induced by the HFD [27] and this was associated with increased GLP-1 production [42]. Probiotic treatments are known to modulate the integrity of the epithelial cell layer [43], and it is possible that a change of intestinal microbiota could modify the nature of microbial-epithelial interactions influencing GLP-1secretion. Although speculative, these hypotheses can be tested in the future using germ free mice [44].

In conclusion our data show for the first time that RSV increases GLP-1 production and requires the GLP-1 receptor to mediate its anti-diabetic effect in HFD-induced diabetic mice. The mechanism(s) through which GLP-1 secretion is restored could be linked to a change in intestinal microbiota and inflammation. Furthermore, our data suggest that RSV, alone or in combination with DPP-4 inhibitors, may represent a new therapeutic approach for enhancing incretin action in the treatment of T2D.
